# Planning the scale up of brief psychological interventions using theory of change

**DOI:** 10.1186/s12913-020-05677-6

**Published:** 2020-08-26

**Authors:** Daniela C. Fuhr, Ceren Acarturk, Marit Sijbrandij, Felicity L. Brown, Mark J. D. Jordans, Aniek Woodward, Michael McGrath, Egbert Sondorp, Peter Ventevogel, Zeynep Ikkursun, Rabih El Chammay, Pim Cuijpers, Bayard Roberts

**Affiliations:** 1 London School of Hygiene and Tropical Medicine; Public Health and Policy, Department of Health Services Research and Policy, 15-17 Tavistock Place, London, UK; 2 Department of Psychology, Koc University, Rumelifeneri, Sarıyer Rumeli Feneri Yolu, Sarıyer/İstanbul, Turkey; 3 Department of Clinical, Neuro and Developmental Psychology, World Health Organization Collaborating Centre for Research and Dissemination of Psychological interventions, Amsterdam Public Health Research Institute, Vrije Universiteit Amsterdam, Van der Boechorststraat 7, 1081 Amsterdam, BT Netherlands; 4 Research and Development Department, War Child Holland, Helmholtzstraat 61, -G Amsterdam, Netherlands; 5 Amsterdam Institute of Social Science Research, University of Amsterdam, Nieuwe Achtergracht, 166 Amsterdam, Netherlands; 6 KIT Royal Tropical Institute, Mauritskade 63, 1092 Amsterdam, Netherlands; 7 Public Health Section, Division of Resilience and Solutions, United Nations High Commissioner for Refugees, Rue de Montbrillant 94, 1201 Genève, Switzerland; 8 National Mental Health Programme, Ministry of Health, Bir Hassan, Beirut, Lebanon; 9 Department of Psychiatry, Faculty of Medicine, Saint Joseph University Beirut, Rue de Damas, B.P.17-5208 Mar Mikhael, Beirut, Lebanon

**Keywords:** Scaling up, Brief psychological interventions, Common mental disorders, Conflict-affected populations

## Abstract

**Background:**

A large mental health treatment gap exists among conflict-affected populations, and Syrian refugees specifically. Promising brief psychological interventions for conflict-affected populations exist such as the World Health Organization’s Problem Management Plus (PM+) and the Early Adolescent Skills for Emotions (EASE) intervention, however, there is limited practical guidance for countries of how these interventions can be taken to scale. The aim of this study was to unpack pathways for scaling up PM+ and EASE for Syrian refugees.

**Methods:**

We conducted three separate Theory of Change (ToC) workshops in Turkey, the Netherlands, and Lebanon in which PM+ and EASE are implemented for Syrian refugees. ToC is a participatory planning process involving key stakeholders, and aims to understand a process of change by mapping out intermediate and long-term outcomes on a causal pathway. 15–24 stakeholders were invited per country, and they participated in a one-day interactive ToC workshop on scaling up.

**Results:**

A cross-country ToC map for scale up brief psychological interventions was developed which was based on three country-specific ToC maps. Two distinct causal pathways for scale up were identified (a policy and financing pathway, and a health services pathway) which are interdependent on each other. A list of key assumptions and interventions which may hamper or facilitate the scaling up process were established.

**Conclusion:**

ToC is a useful tool to help unpack the complexity of scaling up. Our approach highlights that scaling up brief psychological interventions for refugees builds on structural changes and reforms in policy and in health systems. Both horizontal and vertical scale up approaches are required to achieve sustainability. This paper provides the first theory-driven map of causal pathways to help support the scaling-up of evidence-based brief psychological interventions for refugees and populations in global mental health more broadly.

## Background

There is substantial evidence that conflict-affected populations are vulnerable to psychosocial distress and are at risk of considerably higher levels of mental disorders than non-conflict-affected populations [1–3]. Recent estimates suggest that the prevalence of mental disorders is 22·1% at any point in time in conflict-affected populations [2]. High rates of symptoms of common mental disorders are understood as a direct consequence of exposure to violent and traumatic events, and ongoing daily stressors in people’s lives including poor living conditions, impoverishment, unemployment, social isolation and discrimination [1, 3–5].

A large mental health treatment gap (defined as the gap between need and availability of services) has been reported among conflict-affected populations [6–8]. Humanitarian agencies attempt to tackle this treatment gap through the implementation of mental health and psychosocial support (MHPSS) interventions [9, 10]. MHPSS is an umbrella term consisting of individual, group and community activities to improve people’s resilience and mental health outcomes [11]. However, the accessibility and availability of MHPSS interventions in countries neighbouring Syria with large refugee populations (such as Turkey, Lebanon Jordan and Egypt) is limited [12]. A broader range of psychological interventions are being offered in European host countries, however access and utilisation is inadequate as well because of socio-cultural barriers to accessing care including language, cultural understanding of mental health, different interpretation of mental disorder symptoms, stigma and discrimination [3, 12, 13]. Lack of awareness of MHPSS services in European countries have also been reported as barriers to accessing care, in addition to health systems barriers such as long waiting times for treatment, lack of appropriately trained staff (including those who speak Arabic) and lack of interpreters [13].

### Brief psychological interventions and scaling up

Calls to develop and scale up culturally appropriate mental health services globally have been made in the past [14, 15]. However, coverage of mental health services for conflict-affected populations remains low [7, 8]. The World Health Organization (WHO) has responded to this need and advocates for a focused, non-specialised approach and specifically suggests the use of brief, evidence-based psychological interventions for conflict-affected communities as first treatment step [16]. There is evidence that brief psychological interventions are effective in reducing symptoms of mental disorder and psychological distress, and can be integrated into non-specialised (primary) health care in low and middle income countries [15, 17, 18]; this approach also facilitates decentralization of services from tertiary care to the community and is a key policy aim of WHO, partner organisations and governments.

New and innovative brief psychological interventions for conflict-affected populations are currently being implemented and further developed. One of these brief psychological interventions is Problem Management Plus (PM+) [19]. PM+ has been developed by WHO and was specifically designed for adults with psychological distress who are exposed to adversity [20]. It is brief (consisting of five sessions), transdiagnostic, comprises evidence-based techniques of problem solving, stress management, behavioural activation, and strengthening social support; and can be delivered by trained non-specialised providers such as peer workers [19]. PM+ has been proven to be effective in three randomized controlled trials in Pakistan and Kenya (individual PM+ in Pakistan and Kenya, and group PM+ in Pakistan) [21–23]. The Early Adolescent Skills for Emotions intervention (EASE) is a related WHO transdiagnostic intervention [24] that targets young adolescents [25]. It aims to mitigate symptoms of internalizing disorders, such as depression and anxiety. EASE comprises seven group sessions to teach young people skills to enhance psychological coping, complemented by three sessions for caregivers focusing on improving parenting strategies to support their child, and enhance self-care [25].

Brief psychological interventions such as PM+ and EASE are key to closing the treatment gap for conflict-affected populations including Syrian refugees [26] but need to be scaled up. A growing number of frameworks on scaling up health interventions are available in the health literature [27–30]. A prominent one is the WHO ExpandNet framework of scaling up which defines scaling up as “(…) d*eliberate efforts to increase the impact of successfully tested* health innovations so as to *benefit more people and to foster policy and programme development on a lasting basis”* [31]. It describes scaling up as an open system of five elements that interact with one another (the innovation, the user organization, the environment, the resource team and the scaling up strategy), and distinguishes between different types of scaling up (vertical, horizontal, diversification, and spontaneous scaling up, described further in Table 1) [32].

**Table 1:** Four types of scaling up

Types of scaling up	Definition
Vertical scaling up	Institutionalization through policy, political, legal, regulatory, budgetary or other health systems changes
Horizontal scaling up	Expansion (to serve larger or different population groups) or replication (in different geographic sites)
Diversification	Testing and adding a new innovation to one that is in the process of being scaled up
Spontaneous scaling up	Diffusion of the innovation without deliberate guidance

Adapted from WHO/ExpandNet framework of scaling up (Simmons et al., 2007)

While scaling up frameworks show differences between each other and suggest different steps and strategies to scale up [27–30], they have also many commonalities including a common understanding of the attributes of the intervention being scaled up (effectiveness, potential reach, acceptability etc.), identifying and supporting implementers, the selection of an appropriate delivery strategy, understanding and accommodating the characteristics of the adopting community, consideration of the broader socio-political context, and the use of research, evaluation and monitoring data to inform the scale-up process [28]. However, what is missing is practical guidance for countries on what actions to take to bring evidence-based interventions to scale. There is also limited discussion on the essential political and health system requirements which may be the foundation to scale up public health interventions including brief psychological interventions effectively.

Scaling up health services was successfully achieved for a number of global health priorities [33, 34], however, to date, little progress has been made in distributing these interventions to the high numbers of people in need of mental health care in high, low or middle income countries [15]. Barriers to scaling up mental health services have been reported and include competing with other health priorities, financial and human constrains, inflexible health system structures (such as over-centralised care), and poor governance and leadership [14, 15, 35].

### Rationale and objectives

This paper reports findings from the STRENGHTS (Syrian Refugees Mental Health Care Systems) research consortium which evaluates the effectiveness of PM+ and EASE for Syrian refugees in countries neighbouring Syria and major European host countries, and examines its potential for scale up [26]. There are over 5.6 million refugees from the war in Syria, the majority in countries neighbouring Syria such as Turkey, Lebanon or Jordan, with countries in Europe also receiving significant numbers of Syrian refugees [36]. High levels of mental health needs have been reported among Syrian refugees due to their exposure to conflict, violent and traumatic events, and ongoing stressors in their places of settlements [3, 12, 13, 37].

The aim of this study is to unpack pathways for scaling up PM+ and EASE for Syrian refugees. The specific objectives are: (a) to report findings of a cross-country ToC map for scaling up PM+ and EASE for Syrian refugees; (b) to highlight cross-country barriers and facilitators to scale up; and (c) to suggest political and health system changes to make scale up of brief psychological interventions settings a reality.

## Methods

We conducted three one-day ToC workshops in Turkey, the Netherlands and Lebanon which are partner countries of the STRENGHTS research consortium. Turkey and the Netherlands focus on the implementation of group and individual PM+ respectively while EASE is being implemented in Lebanon. We selected these particular countries as study sites for the ToC workshops because of the high number of Syrian refugee populations, and different humanitarian and health system responses and capacity levels. Further information on the characteristics of these settings, and health care entitlements for Syrian refugees in these countries is provided in Appendix A. A range of stakeholders were invited to the ToC workshops based on their expertise on the mental health system and policy in the country, knowledge on the provision of services including PM+ and EASE, and refugee mental health needs (see Table 2). 20–25 participants were invited to the ToC workshops. This number was based on feasibility, and on our expertise in facilitating ToC workshops for other projects**.**

**Table 2:** Stakeholder involvement in Theory of Change workshops

Country /date	Stakeholders	No. of participants
Turkey (Group PM+), November 2018	National and international academics and mental health/conflict researchers from universities in Turkey, the United Kingdom and the Netherlands; personnel from national and international non-governmental organisations such as the UN Refugee Agency, Relief International Turkey, the War Trauma Foundation, and the International Blue Crescent; mental health professionals from local hospitals and community centres; government officials from the Ministry of Health in Ankara	20
The Netherlands (Individual PM+), July 2019	Officials from local non-governmental organisations such as I-psy, Pharos, Veldzicht (Centre for Transcultural Psychiatry) and the War Trauma Foundation; community health care workers; mental health professionals working with Syrian refugees (including one psychiatrist from Syria), conflict and health researchers from the United Kingdom and the Netherlands	22
Lebanon (Early Adolescent Skills for Emotions, EASE), September 2019	National/international academics and mental health/health system’s researchers from universities in Lebanon, the Netherlands and the United Kingdom; officials from local non-governmental organisations such as War Child Holland; officials from international organisations such as UNHCR (the UN Refugee Agency, Geneva and Beirut); community health care workers working with Syrian refugees; mental health service providers; and representatives from the Ministry of Public Health (Mental Health section) in Lebanon	15

### Theory of change (ToC)

ToC is a project planning tool which has increasingly been used in health research in recent years [38, 39]. The overall aim of ToC is to understand the change process of a project or intervention and to map out causal pathways by presenting the sufficient preconditions (or intermediate outcomes) which lead to long-term outcomes and an ultimate impact [38]. It has been used in mental health research as a tool to enhance stakeholder involvement in the implementation and evaluation of psychological interventions [38], and is a useful methodology to unpack complex interventions [40]. ToC has also been used in planning the delivery of mental health care plans and services [41]. It is similar to other project management tools such as logic models, but ToC allows for more flexibility as feedback loops and interactions between causal pathways can be presented in a map [40]. ToC uses specific terminology, described in Table 3.

**Table 3:** Key components of Theory of Change (ToC)

Key components and terminology of ToC	Definition
Impact	The change, real-world impact or vision the project is able to contribute towards.
Long-term outcome	The final and measurable outcome that the project can achieve on its own.
Intermediate outcome	Pre-conditions (or stepping stones) which lead to the long-term outcome in a causal pathway.
Ceiling of accountability	A line (called ceiling of accountability) drawn between the impact and the long-term outcome indicating the level at which implementers stop measuring whether outcomes of the project have been achieved, and therefore stop accepting responsibility of the project’s success or failure.
Assumption	An external condition which must exist for the intermediate outcome on the causal pathway to be achieved.
Intervention	Strategies or activities which bring about intermediate outcomes.
Rationale	Evidence that provides an argument for the selection and importance of each intermediate outcome and long-term outcome, and provides justification for the causal pathway as such.
Indicator	Measures of success aligned with each intermediate outcome, and long-term outcome.

Adapted from DeSilva et al., 2014

The development of the three country-specific ToC maps was informed by several steps. Firstly, we conducted a rapid appraisal on the responsiveness of the mental health system in Turkey, the Netherlands and Lebanon [42]. Syrian refugees in the Middle East and Europe reported high-levels of mental health needs, low contact coverage for MHPSS services, with barriers to care related to language, costs, help-seeking behaviours, lack of awareness, stigma, and negative attitudes towards and by health-care providers [12, 13, 42]. Secondly, we conducted a systematic review to identify barriers and facilitators for scaling up MHPSS interventions for refugees and other populations affected by humanitarian crises [43]. Findings highlighted that scaling-up interventions focused predominantly on integrating services into primary and community care through staff training, task-sharing, and establishing referral and supervision mechanisms. Barriers were reported in a range of ExpandNet framework elements, primarily related to those in the health system rather than broader contextual and structural barriers [43]. Finally, we defined scaling up according to the WHO’s ExpandNet framework of scaling up as outlined above, and used the ExpandNet framework to guide our work.(31)Three separate ToC workshops were then held in Turkey, the Netherlands and Lebanon.

All country-specific ToC maps were developed with stakeholders participating in the workshops (see Table 2), and were further contextualised and finalised through small group discussions with STRENGHTS country partners. No quotes were taken from ToC workshop participants. The cross-country ToC map was developed iteratively by STRENGTHS research consortium members who participated in the ToC workshops. Commonalities and differences between country-specific ToC maps were colour-coded, and key components of a common policy/finance pathway and health system/service pathway were identified and mapped on two distinct causal pathways.

## Results

**Fig. 1:**
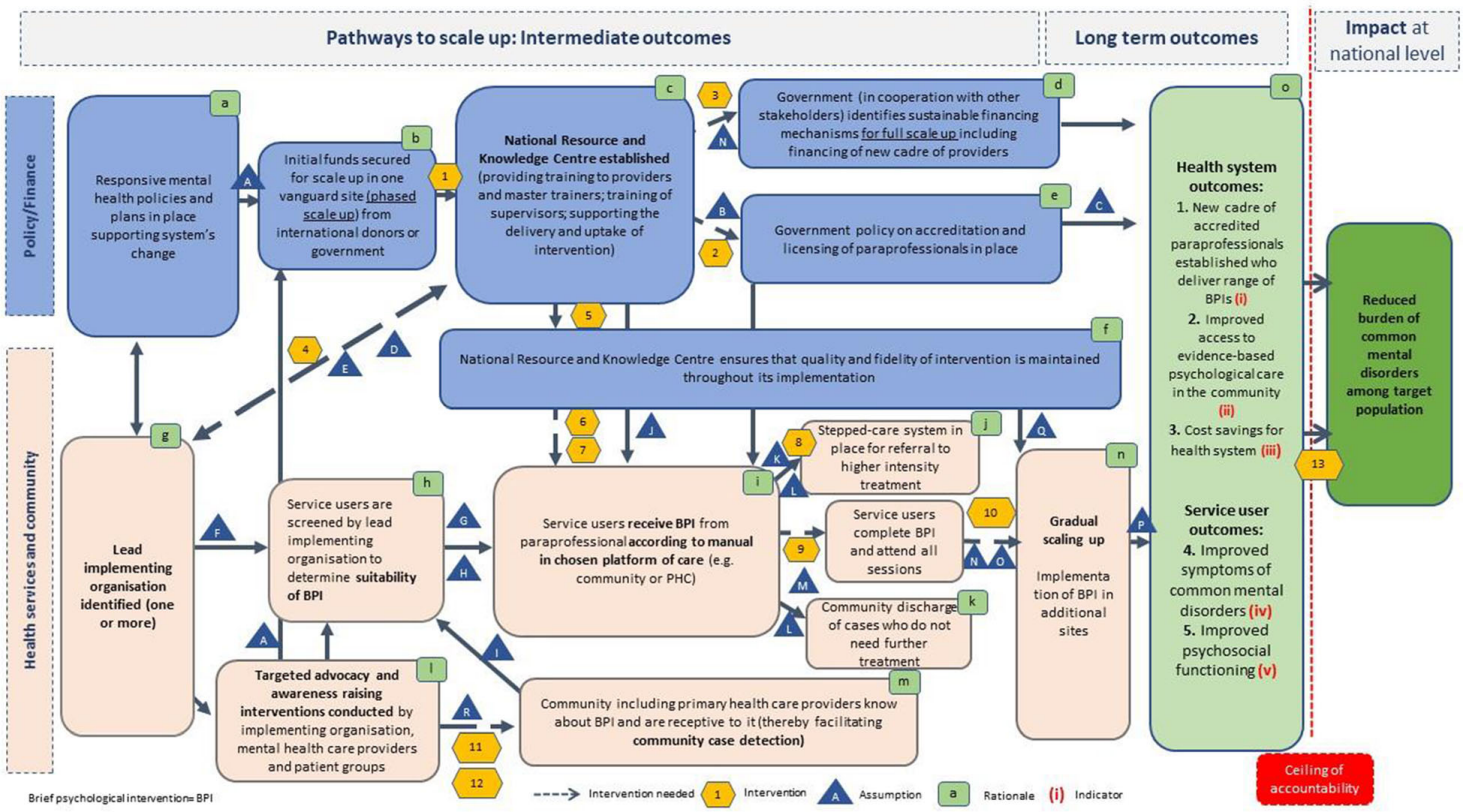
Cross-country ToC map (own figure). Note: Legends for interventions, assumptions, rationale and indicators are provided in the online annex (Appendix B)

The cross-country ToC map on scaling up brief psychological interventions for refugees is presented in Fig. 1 while legends describing interventions, assumptions, rationales, and example indicators for long-term outcomes are included in Appendix B. The country-specific ToC maps and legends for Turkey, the Netherlands and Lebanon are included in the annexes (Appendix C-E). Key differences between the country-specific ToC maps are outlined in Appendix F.

Key elements of scaling up were established (health services and the community; policy and finance) which are represented in two distinct causal pathways. Thirteen interventions (intervention 1–13) and 18 assumptions (assumption A-R) were identified. Intermediate outcomes were supported by 15 rationales (rationale a-o). Key assumptions and interventions are included in the description of the pathways to scale up further below; the remainder of assumptions and interventions are included Appendix B.

### Pathways to scale up

An essential pathway to scale up brief psychological interventions was the ‘Policy and Finance’ pathway. The availability of ‘Responsive mental health policies and plans supporting systems change’ was the earliest change identified by stakeholders on that pathway. ‘Initial funds’ (either from international donors or the government) was identified as imperative to support a phased scale up of the intervention in one vanguard/pilot site. Advocacy efforts by the lead implementing organisation, health professionals and patient groups to obtain sufficient funding for scale up in the vanguard site was identified as essential underlying assumption (assumption A). Stakeholders strongly supported the establishment of a ‘National Resource and Knowledge Centre’ that has the mandate to provide training to paraprofessionals and master trainers. The National Resource and Knowledge Centre was expected to support the delivery of the psychological intervention more broadly ensuring that ‘Quality and fidelity of the intervention is maintained throughout its implementation’. The government in cooperation with other national stakeholders would identify ‘sustainable financing mechanisms required for full scale up’ which would include the establishment and financing of a new cadre of paraprofessionals. These paraprofessionals would be employed to deliver (a range of) brief psychological interventions. This is built on the premise that the initial scale up in the vanguard site has proven to be beneficial for service users and the health system (assumption N); and that scale up is supported through system’s change (intervention 3). The establishment and employment of a new cadre of paraprofessionals was viewed as an important requirement and building on a policy of accreditation and licensing of these paraprofessionals. There was an assumption that the National Resource and Knowledge Centre would need to work together with a higher education institute to provide an accreditation certificate to paraprofessionals who complete a course on brief psychological interventions (assumption B).

The second pathway to scale up was the ‘Health services and community’ pathway which is influenced and dependent on the ‘Policy and Finance’ pathway. The earliest intermediate outcome of the ‘Health services and community’ pathway was the identification of one or several implementing organisation(s) that would lead the delivery of the intervention in the vanguard site. The National Resource and Knowledge Centre would be expected to actively strengthen the implementation capacity of the implementing organisation through provision of skills training, personnel, logistics and supplies (intervention 4). An equitable partnership and formal leadership structure was assumed between these two organisations (assumption E). The implementing organisation would be responsible to ‘identify/screen service users to determine suitability of the intervention’ and would subsequently ‘deliver the intervention to service users by employing paraprofessionals’. The implementing organisation would equally be engaged in ‘targeted advocacy and awareness raising together with mental health care providers and patient groups’ to influence outcomes on the policy/finance pathway (i.e. leverage of funds), and to increase ‘knowledge about the intervention in the community thereby facilitating community case detection’ and increasing treatment demand (assumption R). Community case detection was built on the premise that key actors in the community who are in close contact with the target audience would receive a short training on mental health and the use of a community case detection tool to identify persons in need for a brief psychological intervention (intervention 12). Paraprofessionals delivering the intervention would be supervised by the National Resource and Knowledge Centre via mental health professionals (intervention 7). The implementation of a brief psychological intervention was understood as first step requiring a ‘stepped-care system to be in place’ for service users who do not respond to a brief psychological intervention only. A treatment protocol (assumption L) would support stepped-care guiding paraprofessionals on when service users would be ‘discharged into the community’ or being ‘referred to higher intensity treatment’. Stepped-care would be supported by implementing a model of collaborative care in the mental health system (intervention 8) and would assume the availability of mental health professionals in tertiary care to provide higher intensity treatment (assumption K). The National Resource and Knowledge Centre would employ quality standard checks ensuring that the brief psychological intervention is delivered according to its manual; this would be done by regular inspections of the implementing organisation(s) and by supervisors to monitor fidelity (intervention 9). After successful implementation of the intervention in the vanguard site, the intervention would be expanded to other sites through ‘gradual scale up’. Expansion of the intervention to additional sites was assumed to build on ‘positive health and system outcomes’ in the vanguard site (assumption N) and close collaboration between the implementing organisation and the National Resource and Knowledge Centre. Expansion would be based on knowledge of the size of the population in need to project current and future demand of the intervention (assumption O). For wider use of the intervention and its use by other mental health professionals there is a need to incorporate the intervention into national or international mental health guidelines (intervention 10).

Both pathways to scale up lead to distinct long-term outcomes (three health system outcomes, and two service user outcomes specifically) and an envisaged impact. An underlying assumption for the realisation of long-term outcomes was an ‘outcome and monitoring system to be in place’ operated by the National Resource and Knowledge Centre. This would include a feedback and complaint system allowing service users to voice concerns to the lead implementing organisation(s) and the National Resource and Knowledge Centre. ‘Reduced burden of common mental disorders among target population’ was identified as broader vision or impact that the scaling up of a brief psychological intervention may be able to contribute towards. To enable this, a ‘multiplication of delivery platforms, and delivery of the intervention across different levels of the health and social care system’ was suggested (intervention 13).

## Discussion

Scaling up evidence-based psychological interventions is key to closing the treatment gap for mental disorders. While promising psychological interventions for conflict-affected populations exist, [18, 44] there is limited practical guidance of how these interventions can be taken to scale [8]. The WHO’s ExpandNet framework of scaling up health interventions recommends that the scale up of an intervention should be developed through a participatory process involving key stakeholders [29, 31]. However, it does not provide methodological guidance of how to do this.

The cross-country ToC map highlights six key lessons for scaling up brief psychological interventions such as PM+ and EASE. First, the two pathways to scale up, ‘finance and policy’ and ‘health services and the community’ highlight the need for both vertical and horizontal scale up. Both horizontal and vertical scaling up are imperative for a sustainable scale up; expansion or replication is understood to be insufficient on its own [45]. Our ToC map shows the interdependency between both pathways and both types of scaling up, highlighting that expansion of an intervention (horizontal scaling up) by the implementing organisation may be prone to failure if it is not supported by political, regulatory or other health system changes. The second lesson is that brief psychological interventions may best be integrated in a stepped-care model which may make care more cost-effective [46]. Stepped-care is realised if the most effective yet least resource intensive treatment is delivered to patients initially [47]; more intensive treatment is only offered if patients do not benefit from the previous treatment step. This model of care is built on a clear rational for clinical decision making involving guidelines, [47] and should ideally be supported by collaborative care including a case manager. Third, brief psychological interventions may only facilitate access to care if they are developed with scaling up in mind. This requires awareness of potential implementation barriers during the early phase of intervention development as any barrier to accessing care may be amplified during scale up. These may include structural barriers such as lack of transport, inability to obtain convenient appointment times, lack of childcare options at provider and/or attitudinal barriers including fear of stigma and discrimination. Some of these barriers such as transport problems may be beyond the programme of care to resolve on its own, however, methods should be considered for mitigating their negative effect on service uptake. Increase in service coverage may also require alignment of the intervention into routine practice of humanitarian agencies and other sectors such as education or protection which may offset some barriers to accessing care. It may also be important to look at demand side factors including stigma to increase help-seeking. The fourth lesson was the need for a National (or centralised) Resource and Knowledge Centre in countries. This can be understood as centre of excellence for brief psychological therapies. It would seek to ensure quality control including the delivery of accredited training and supervision and to prevent the unregulated delivery of the intervention (or the diffusion of the intervention without deliberate guidance and training) [45]. Fifth, we learned that a phased scale up may be beneficial. This refers to the initial expansion of the intervention in one pilot or vanguard site only. This has the advantage that early teething problems during implementation can be recognised and adjustments taken [48]. It may also offer the opportunity to streamline mental health with other activities provided in the health or social care system, and may allow for lasting institutional capacities to be built between the implementing organisation(s) and the National Resource and Knowledge Centre. Finally, we have seen that scaling up may be facilitated if it is built on responsive health care policies and plans which recognise the need to scale up mental health services for vulnerable populations. Scaling up brief psychological interventions may imply structural health system changes in some countries such as the implementation of a stepped-care system. This may require a fundamental shift of service delivery in some countries, including changing roles for a variety of health care providers and training. However, these health system changes may ultimately strengthen the health systems, and may make better use of limited human and financial resources.

### Limitations

While effectiveness and cost-effectiveness of PM+ has been demonstrated with other adversity-affected populations [21–23], the effectiveness of PM+ and EASE in Turkey, the Netherlands and Lebanon is still being evaluated through ongoing randomised controlled trials [26]; therefore, in our ToC workshops we assumed effectiveness and scalability of these interventions with Syrian refugees. We found ToC to be a valuable tool outlining how change occurs, however, ToC is unable to investigate the reasons behind the actual change process. Future research could explore reasons for the selection of assumptions or interventions in real scenarios, or explore the change pathway using in-depth qualitative research. We invited a range of multiple stakeholders building on our expertise in the study countries; however, future work could look at developing guidance for countries on the essential actors which need to be involved in ToC scaling up mapping. We invited refugees from Syria to our ToC workshops but only two were able to participate. Strong efforts should therefore be made to increase the number of people with lived experience for future workshops on the topic. Further, our scenarios focus on Syrian refugees specifically rather than other conflict-affected populations (such as internally displaced persons and non-displaced populations exposed to other aspects of adversity). We recommend that future studies look at scaling up MHPSS interventions for these populations and contexts. Finally, our cross-country ToC map was developed to provide an initial roadmap for countries on how brief psychological interventions can be taken to scale. Parts of it may become obsolete as national circumstances change, however, the flexible approach of ToC can allow the map to be adapted to take account of future changes. The ToC map can also be used for monitoring and evaluation but this would require intermediate outcomes to be aligned with indicators to measure change in practice. While we have provided example indicators for the long-term outcomes of our cross-sectional ToC map, we have not developed indicators for intermediate outcomes. Monitoring and evaluation of scaling up is encouraged but this relies on the availability of health service data to estimate effectiveness coverage which may not be readily available in many countries.

## Conclusion

Conducting ToC workshops on scaling up brief psychological interventions such as PM+ and EASE provided valuable insights for policy, practice, and research. Our paper maps out pathways to scaling up from three diverse Middle-Eastern and European country settings and highlights six key lessons to support policy and health system reforms. We found ToC to be a valuable research tool; its methodology helped to unpack the complexity of scaling up and provided a structured means of working together with key stakeholders. To the best of our knowledge, this paper provides the first theory-driven map of causal pathways to help support the scaling-up of evidence-based brief psychological interventions.

## Supplementary information

**Additional file 1.** Annex A: Characteristics of Syrian refugees and their health care entitlements in the study countries. Annex B: Cross-country ToC map and legends. Annex C: ToC map and legends for Turkey. Annex D: ToC map and legends for the Netherlands. Annex E: ToC map and legends for Lebanon. Annex F: Main differences between individual ToC maps.

## Data Availability

All data generated or analysed during this study are included in this published article.

## Abbreviations

EASE: Early Adolescent Skills for Emotions
MHPSS: Mental Health and Psychosocial Support
PM+: Problem Management Plus
ToC: Theory of Change
WHO: World Health Organization
