# Supplementary material for: Planning the scale up of brief psychological interventions using theory of change
Source: BMC Health Serv Res. 2020 Aug 26;20:801. doi: 10.1186/s12913-020-05677-6 (PMC7449040; doi:10.1186/s12913-020-05677-6)
Supplement: Supplementary file 1 — Additional file 1. Annex A: Characteristics of Syrian refugees and their health care entitlements in the study countries. Annex B: Cross-country ToC map and legends. Annex C: ToC map and legends for Turkey. Annex D: ToC map and legends for the Netherlands. Annex E: ToC map and legends for Lebanon. Annex F: Main differences between individual ToC maps. [file 12913_2020_5677_MOESM1_ESM.docx]

## Annex A: Characteristics of Syrian refugees and their health care entitlements in the study countries

|  | **Turkey** | **The Netherlands** | **Lebanon** |
| --- | --- | --- | --- |
| **Number of Syrian refugees/asylum seekers** | 3.587.566 registered with the Turkish government. | Between April 2011 and June 2017, 33,897 Syrians sought asylum in the Netherlands. | 914,648 registered with the UNHCR, 1,500,000 estimate |
| **Gender (%) and mean age** | 54% male;  44% aged under 18 | Of the 27,971 Syrian nationals given a residence permit in 2016: 61% were male and 36% aged under 18 | 48% male;  56% aged under 18 |
| **Settlement types** | Predominantly urban (2% living in government camps) | Urban | Most live in urban centres, villages and informal settlements. |
| **Legal status** | Syrians are entitled to residency once they register for temporary protection with the Provincial Directorate of Migration Management. | Refugees are given a temporary residence permit for five years. After which, if they still require protection and complete a Dutch integration exam, they can be given permanent residency. | Limited legal status. Only 22% of Syrians aged over 15 years of age have legal residency in Lebanon. |
| **Lead health agencies and service providers** | Ministry of Health, municipal government, NGOs | Ministry of Health (VWS), Central Agency for the Reception of Asylum Seekers (COA), municipal government, NGOs | Ministry of Public Health, UNHCR, UNICEF, NGOs |
| **Health care entitlements** | Registration for temporary protection affords Syrians legal access to health services. | Syrians with a residence permit are entitled to the same health care as Dutch citizens | Registered Syrians can access primary care, dispensaries and hospitals – but may need to pay for consultations and prescriptions. |

Source: UNHCR (2019) Vulnerability Assessment of Syrian Refugees in Lebanon 2019; UNHCR (2020). Syria Regional Refugee Response. <https://data2.unhcr.org/en/situations/syria>; World Food Programme 2019. Comprehensive Vulnerability Monitoring Exercise Round 3

## Annex B: Cross-country ToC map and legends


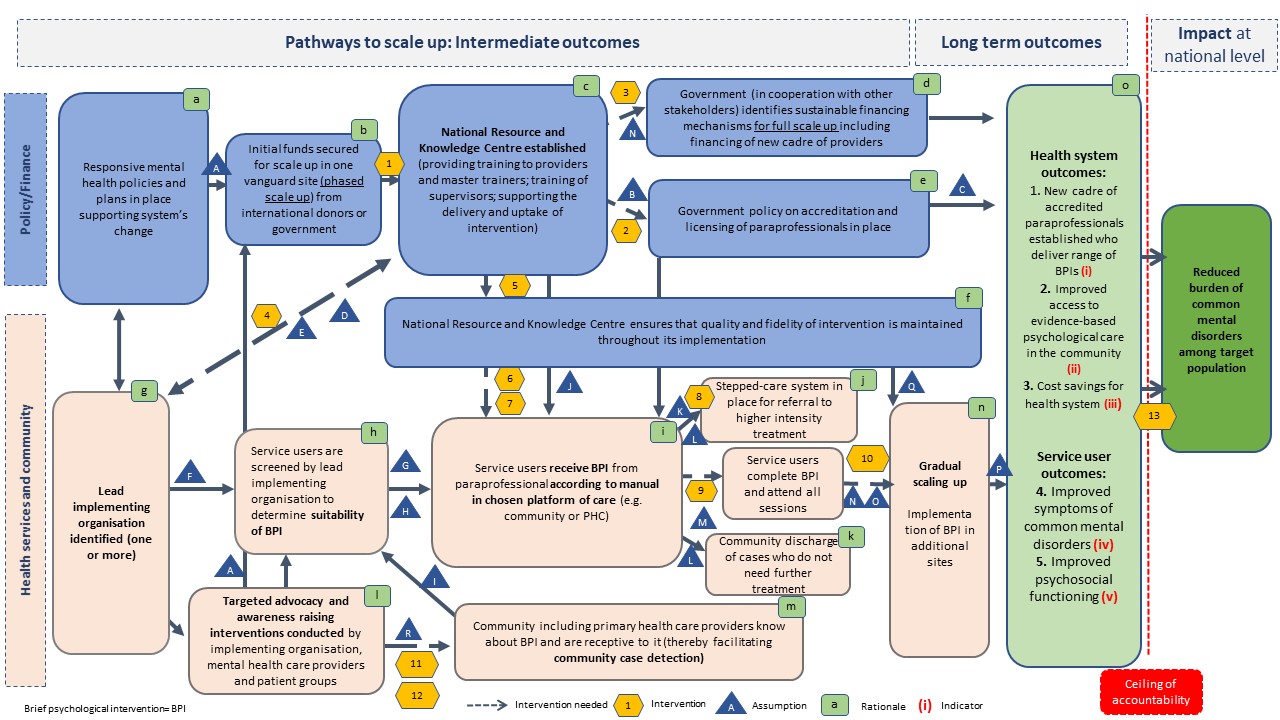


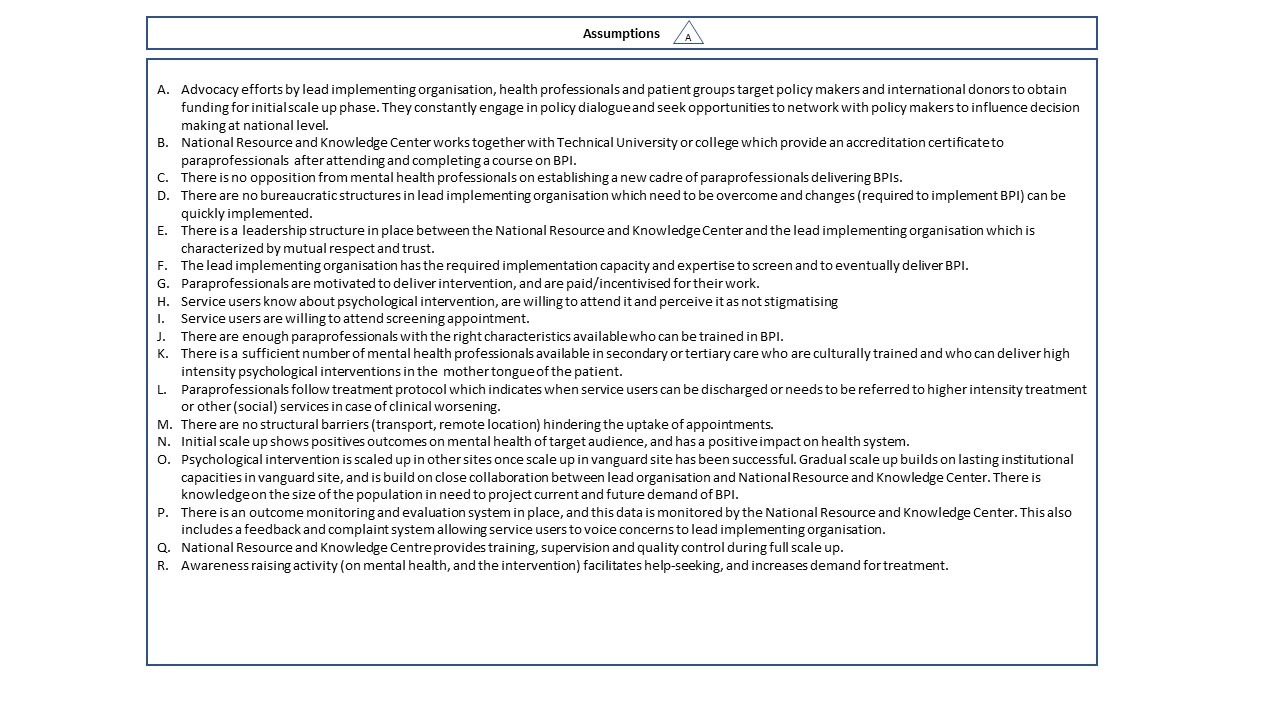


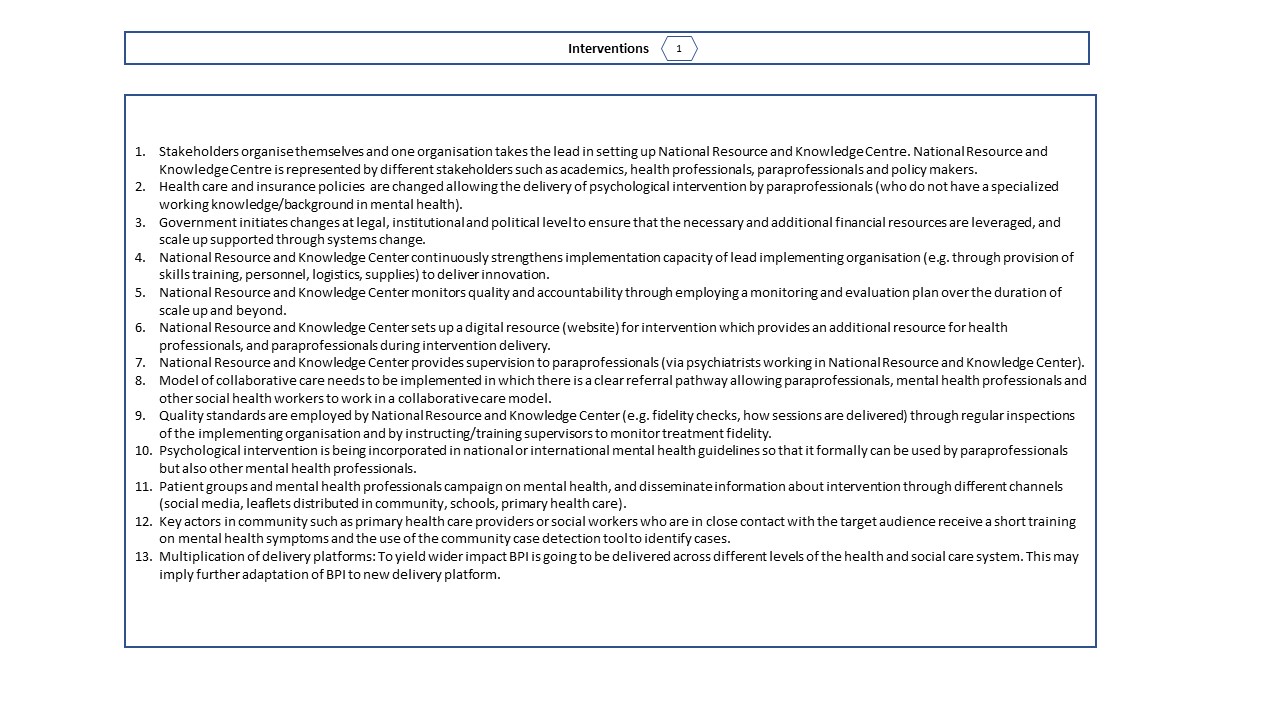


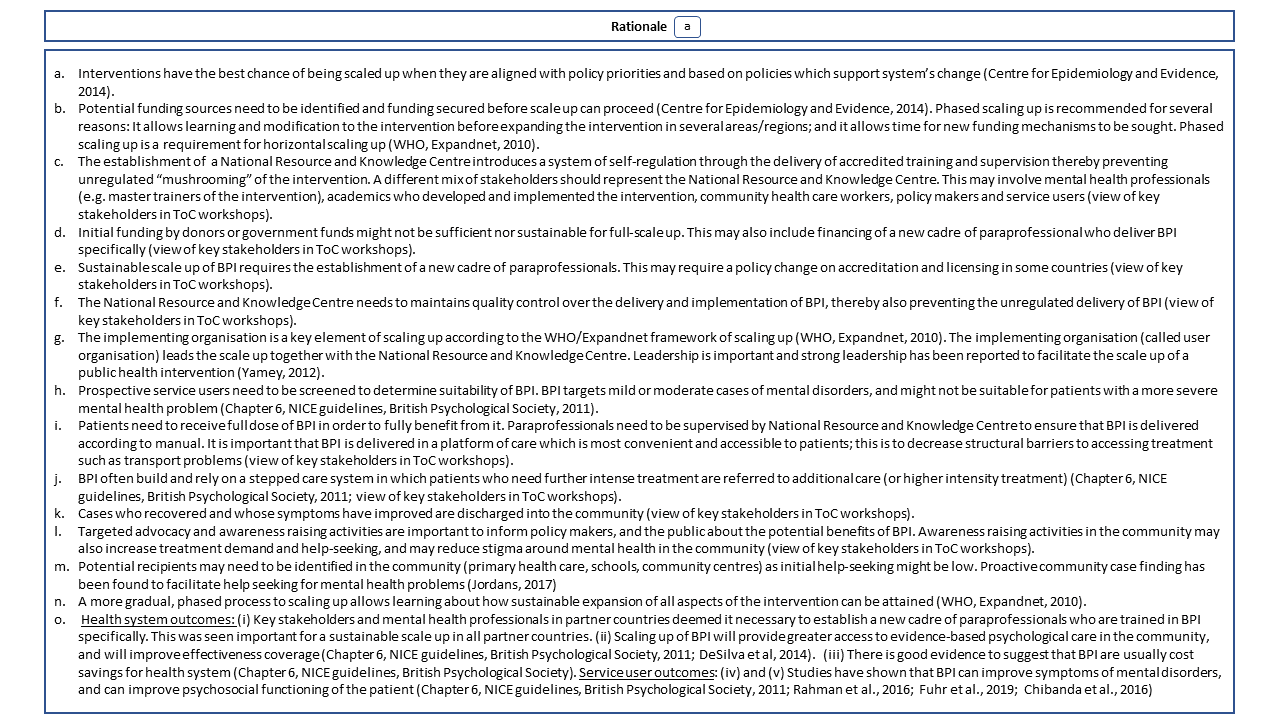


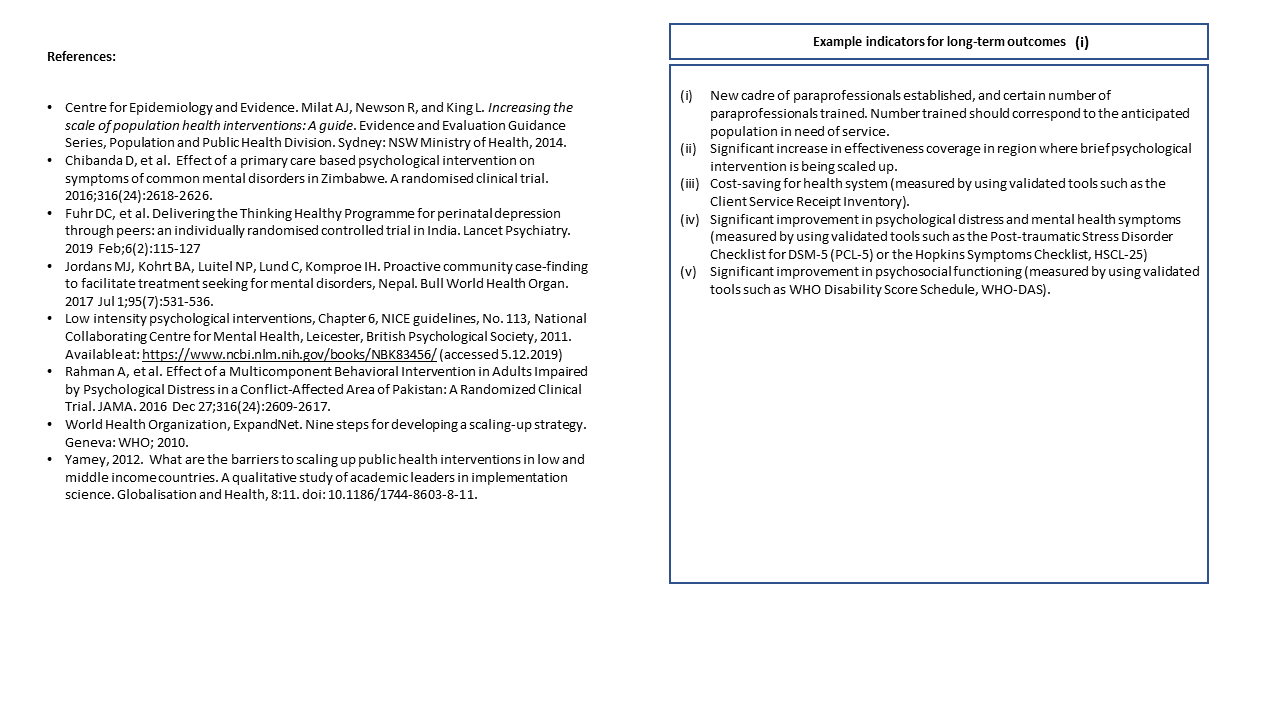
Note: We did not assign indicators for intermediate outcomes as the cross-country ToC map is not yet intended to monitor or measure success of the scaling up pathways; to exemplify the use of indicators we have developed indicators for long-term outcomes only. In routine monitoring, these would need to be specified further by assigning them with a time-frame of when success would be expected.

## Annex C: ToC map and legends for Turkey


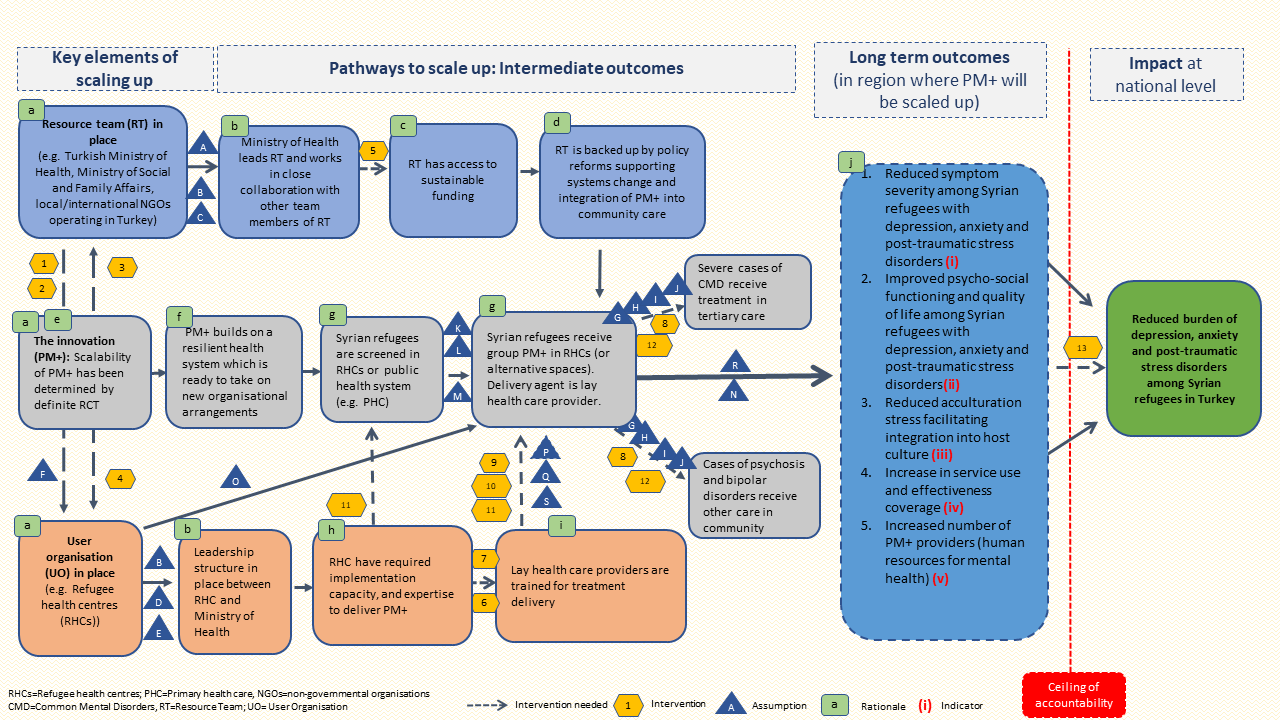


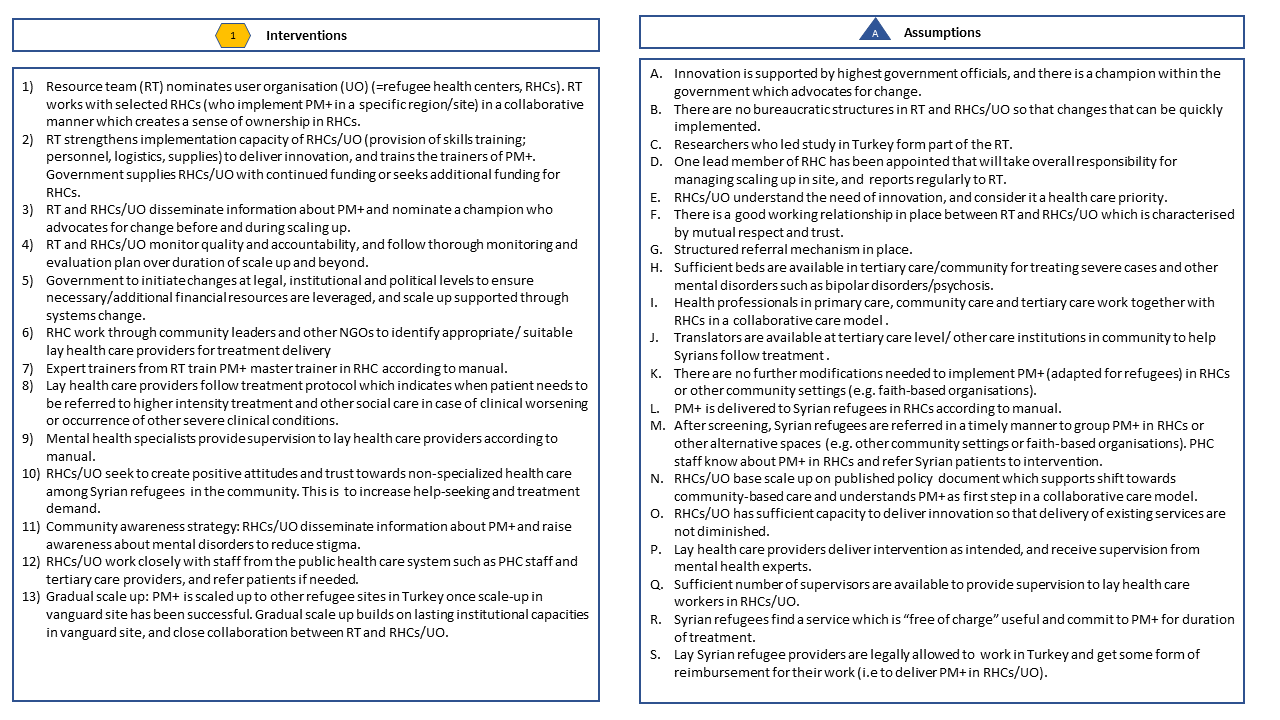


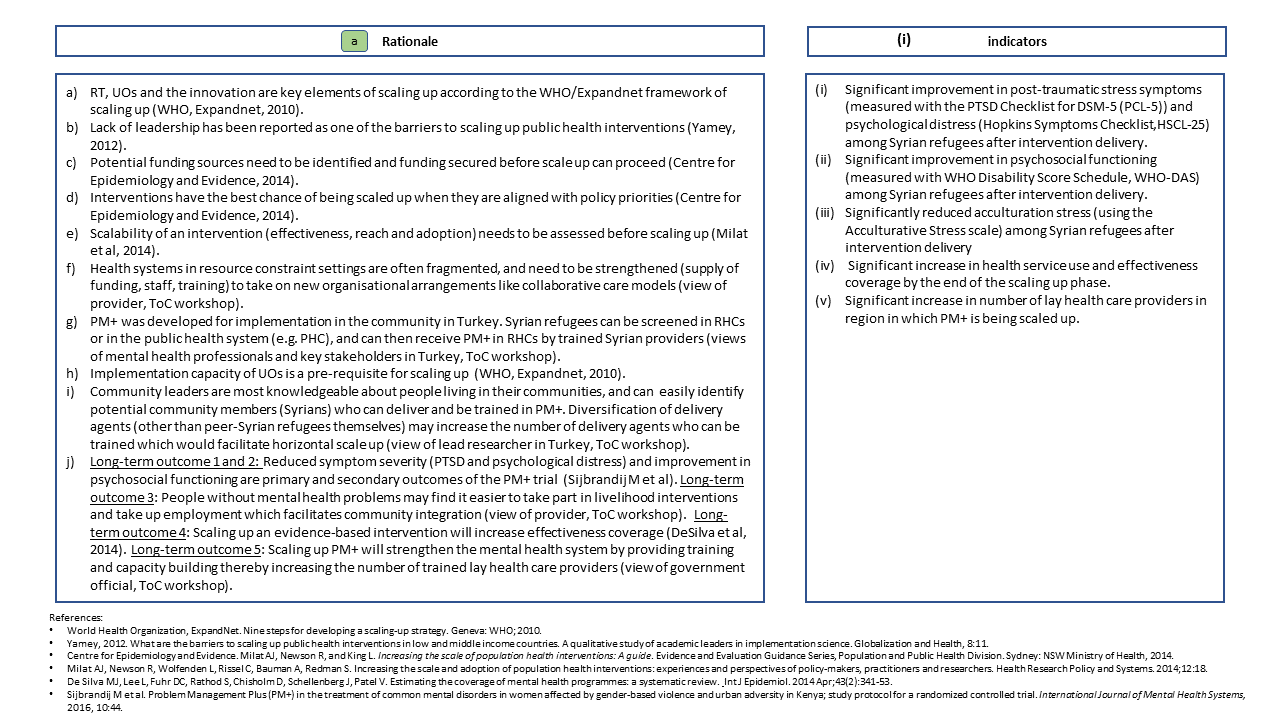


Note: We did not assign indicators for intermediate outcomes as the cross-country ToC map is not yet intended to monitor or measure success of the scaling up pathways; to exemplify the use of indicators we have developed indicators for long-term outcomes only. In routine monitoring, these would need to be specified further by assigning them with a time-frame of when success would be expected.

## Annex D: ToC map and legends for the Netherlands


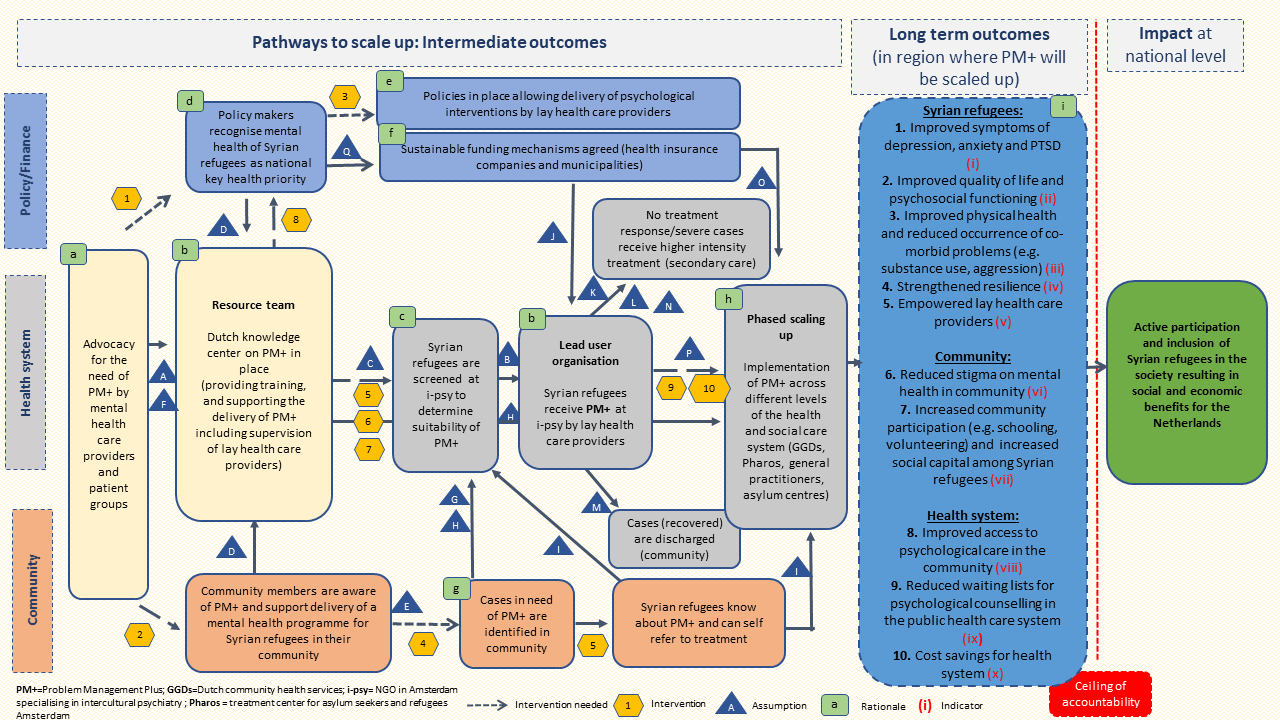


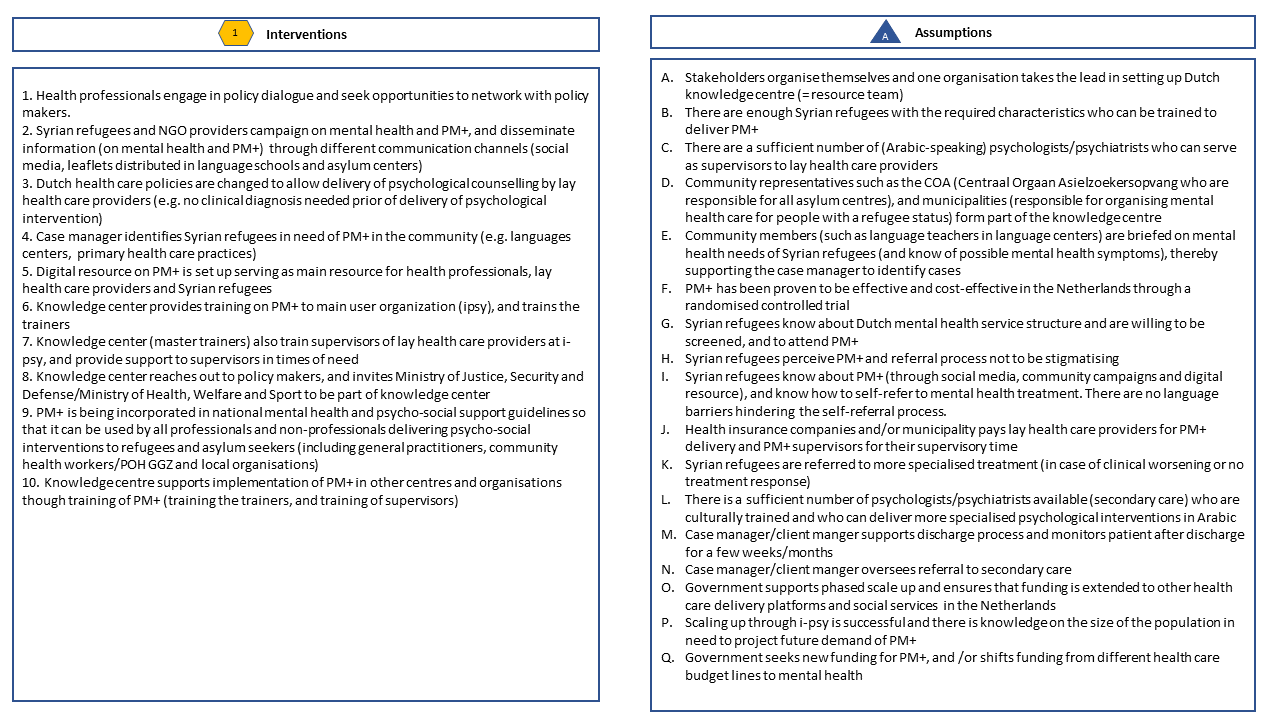


##
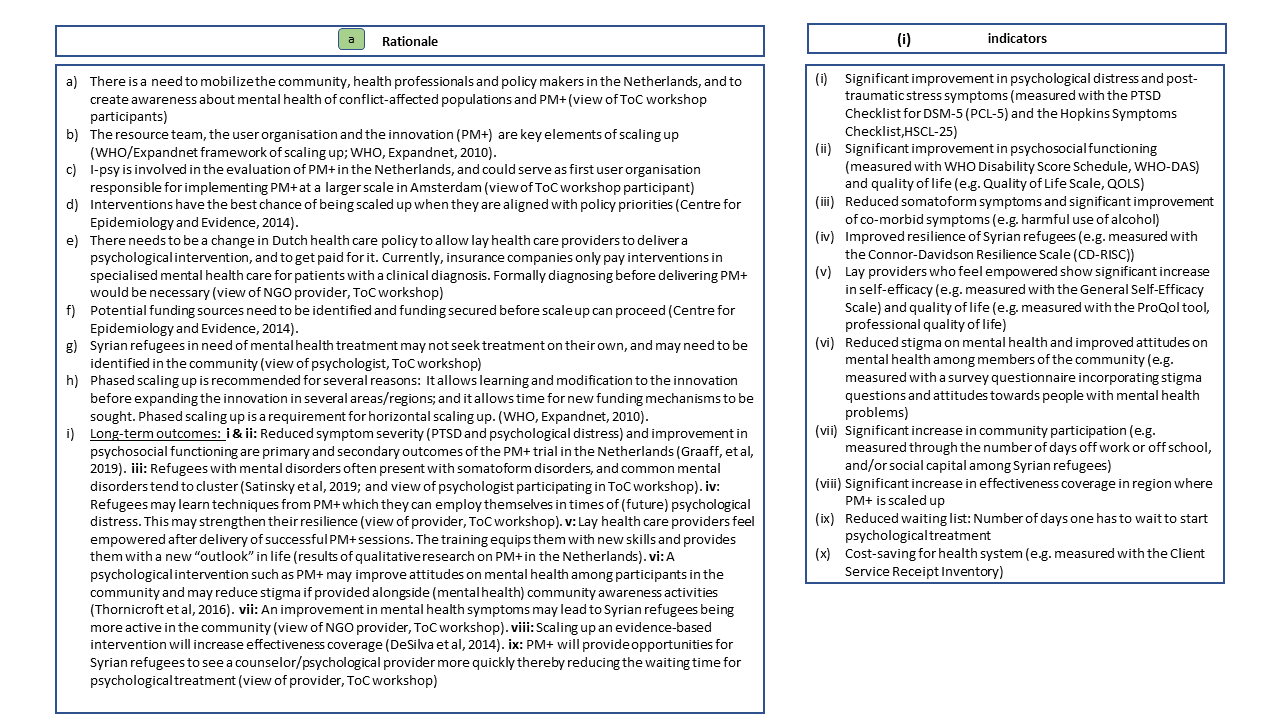


Note: We did not assign indicators for intermediate outcomes as the cross-country ToC map is not yet intended to monitor or measure success of the scaling up pathways; to exemplify the use of indicators we have developed indicators for long-term outcomes only. In routine monitoring, these would need to be specified further by assigning them with a time-frame of when success would be expected.

##
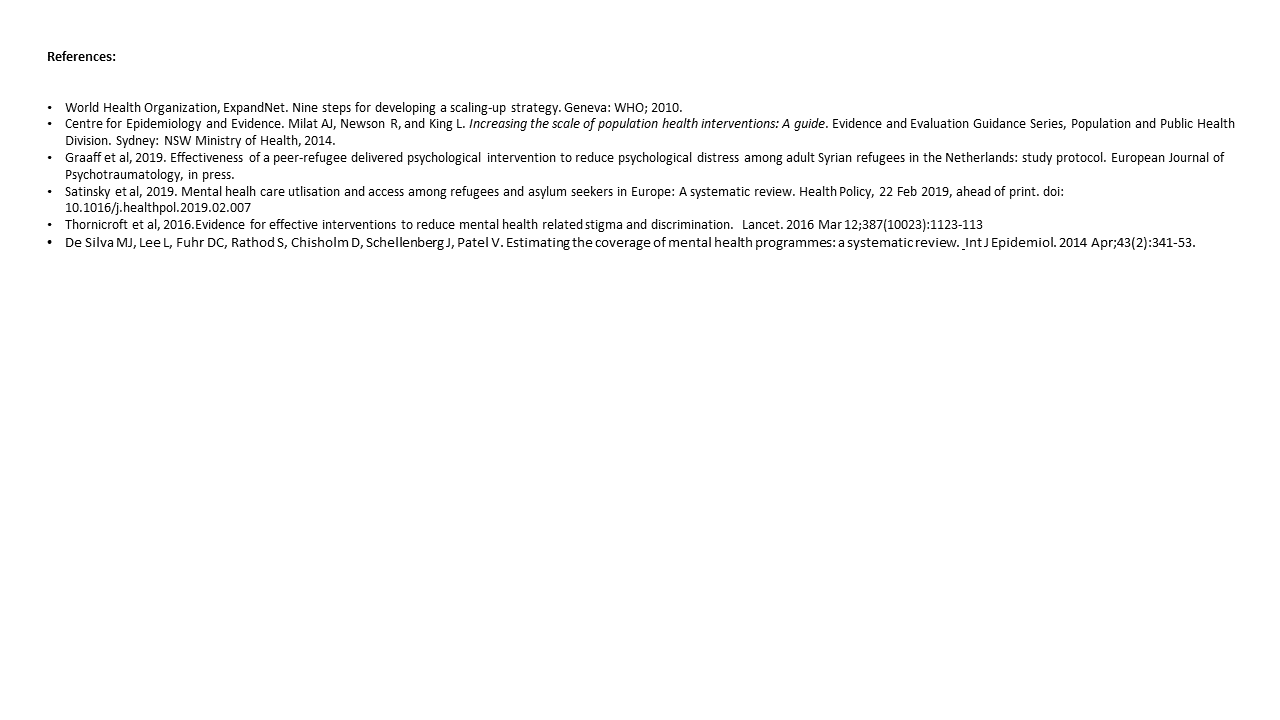


## Annex E: ToC map and legends for Lebanon


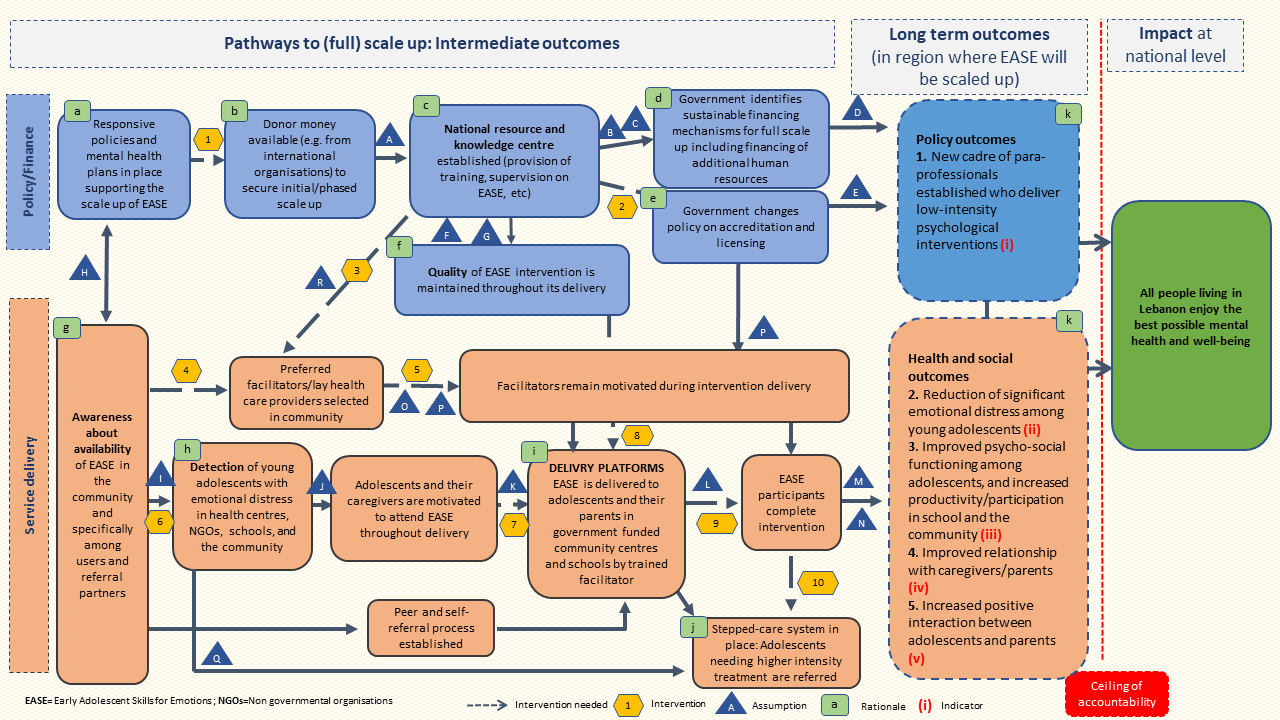


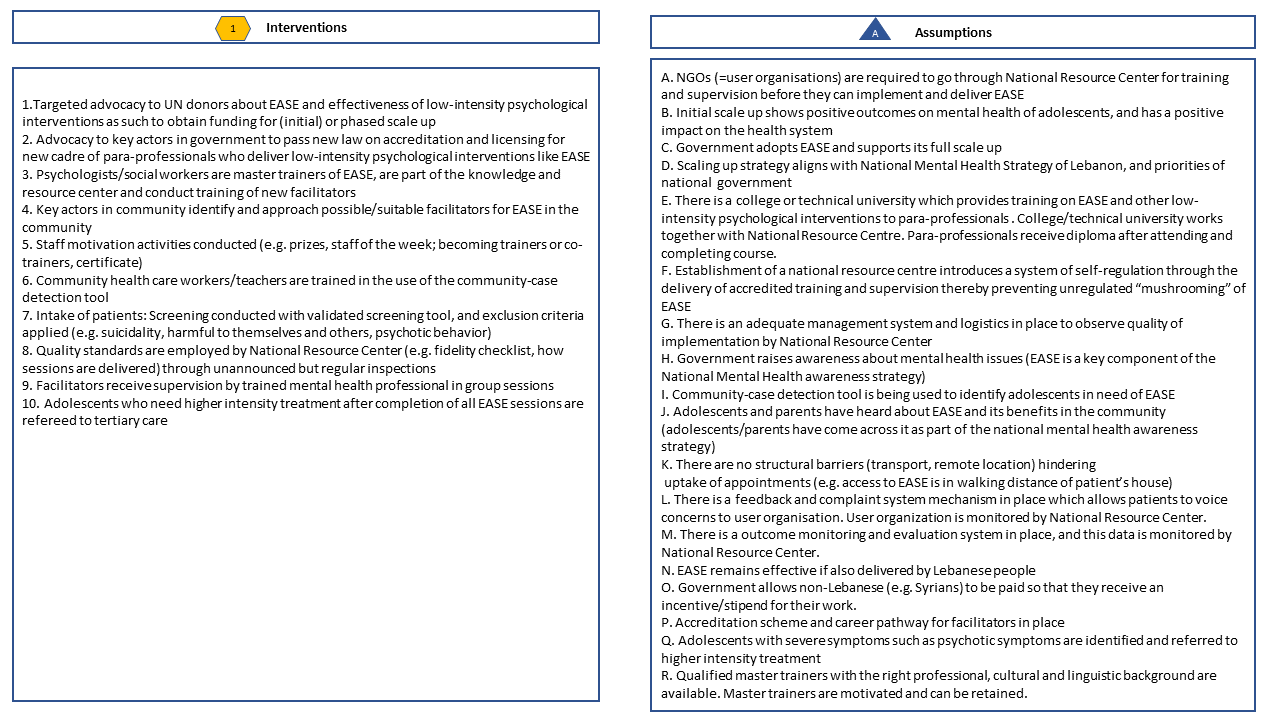


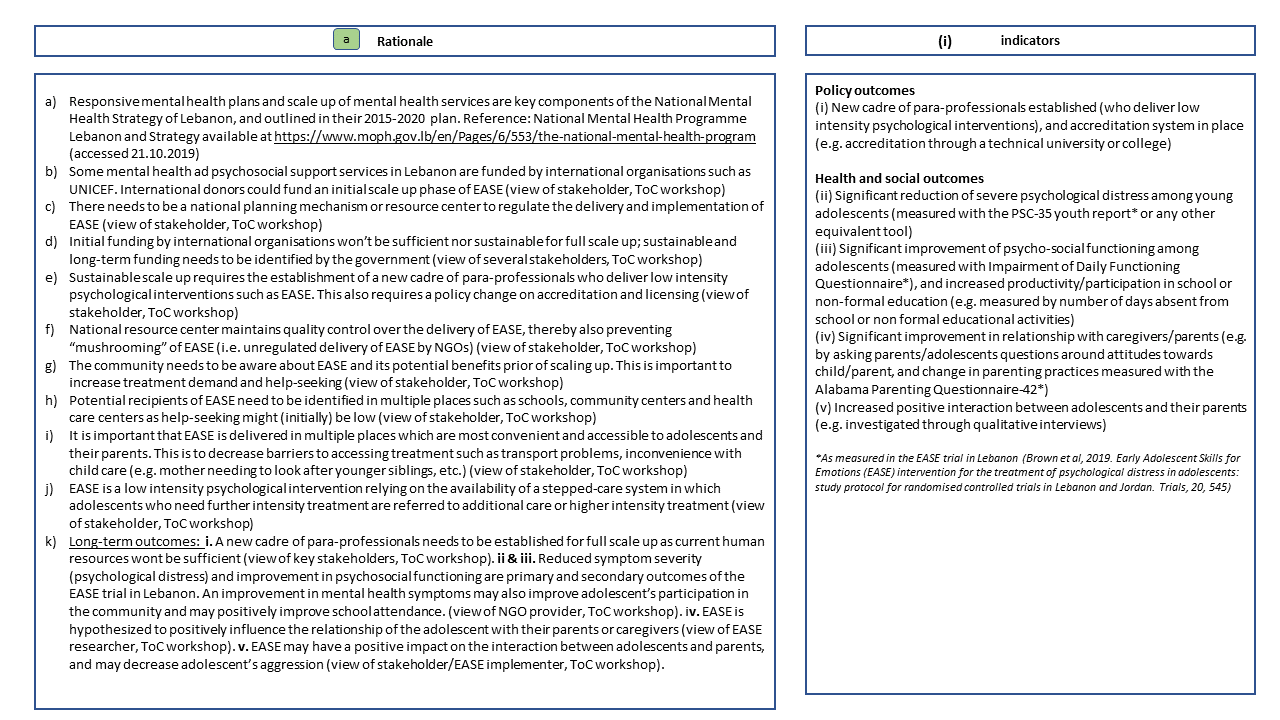


Note: We did not assign indicators for intermediate outcomes as the cross-country ToC map is not yet intended to monitor or measure success of the scaling up pathways; to exemplify the use of indicators we have developed indicators for long-term outcomes only. In routine monitoring, these would need to be specified further by assigning them with a time-frame of when success would be expected.

## Annex F: Main differences of individual ToC maps

Country-specific ToC maps were largely similar to each other (see annexes B-D), identifying overlapping pathways of scaling up, assumptions and interventions. One area of difference was related to the agency taking a lead in organising services and scale up (also highlighted in Table 1). For example, in the Netherlands, scaling up psychological interventions may be the responsibility of different stakeholders such as the national or local governments, health insurers or municipalities. In Lebanon, scale up in urban areas may involve both government and humanitarian agencies. However, humanitarian NGOs may predominantly take the lead in scaling up services in selected geographical areas (with their own funding leaving other areas underserved). In Turkey, the majority of Syrian refugees live in urban areas, and there are a few camps only. Humanitarian agency may be less involved in scale up services in Turkey, with the government taking a leading role there. Another difference may relate to health insurance and coverage of costs during scale up in our study countries. Syrian refugees receive free health insurance in the Netherlands; however, health insurance may not pay for psychological treatment if it is provided by a lay health care provider, and if no psychiatric diagnosis is made in advance of treatment. This may hamper scale up, and expansion of a collaborative stepped-care system in the Netherlands.
